# Children atopic dermatitis: Diagnosis, mimics, overlaps, and therapeutic implication

**DOI:** 10.1111/dth.15901

**Published:** 2022-10-13

**Authors:** Maddalena Napolitano, Gabriella Fabbrocini, Fabrizio Martora, Lucia Genco, Matteo Noto, Cataldo Patruno

**Affiliations:** ^1^ Department of Medicine and Health Sciences Vincenzo Tiberio University of Molise Campobasso Italy; ^2^ Section of Dermatology – Department of Clinical Medicine and Surgery University of Naples Federico II; ^3^ Department of Health Sciences University Magna Graecia of Catanzaro Catanzaro Italy

**Keywords:** atopic dermatitis, diagnosis, therapy

## Abstract

Atopic dermatitis (AD) is a chronic inflammatory, itching skin with a significant psychosocial impact on patients and relatives. In adults and adolescents besides flexural eczema, head and neck eczema, and hand eczema, which are the most frequent clinical phenotypes (84.9% and 84.2%, respectively), there are also other possible presentation such as, portrait‐like dermatitis (20.1%), diffuse eczema (6.5%), eczema nummulare‐like (5.8%), prurigo nodularis‐like (2.1%) and erythrodermia (0.7%). Diagnosis can be easy due to the typically distributed eczematous lesions, albeit with age‐related differences, However, it is also extremely heterogeneous in severity, course, and sometimes particular clinical features. Currently, there are no better diagnostic criteria than an experienced dermatologist for the diagnosis of AD. Misdiagnosis and delayed treatment will have an impact not only on the child's physical health, but also and especially on the child's psychological health. The aim of our review was to group the main differential diagnoses in pediatric age where the diagnosis can often hide many pitfalls.

## 1 INTRODUCTION

Atopic dermatitis (AD) is a chronic inflammatory, itching skin with a significant psychosocial impact on patients and relatives.^1^, ^2^ It usually starts in infants or children (early‐onset AD) and may represent the initial step of the so‐called “atopic march” in which AD is followed by other atopic diseases (asthma, rhino conjunctivitis, and eosinophil esophagitis).^3^ In most cases, AD heals during childhood; however, it may persist in older ages. In the majority of adolescent or adult patients the disease lasts since younger ages (persistent AD), but it can also begin during the adolescence or adulthood (adolescent‐onset AD or adult‐onset AD).^4^, ^5^ A separate subgroup of elderly‐onset AD (aged ≥60 years) has recently been described.^6^ The diagnosis is considered easier in childhood than in older ages due to a more polymorphous clinical features in the adult. Notwithstanding, the clinical phenotypes of AD may be sometimes atypical also in the younger.

In this narrative review, we evaluated the diagnostic challenges of AD in pediatric age (0–11 years) and its management.

### 1.1 Epidemiology

Since the 1970s, AD incidence has increased 2‐ to 3‐fold in industrialized nations, impacting approximately 15% to 20% of children, 5% to 20% of adolescents, and 1% to 3% of adults.^3^ However, the International Study of Asthma and Allergies in Childhood (ISAAC) study, reported that the prevalence widely varies depending on geographical areas.^7^, ^8^, ^9^

Indeed, in the age group 6–7 years, it ranges from 0.9% in India to 22.5% in Ecuador, with new data showing high values in Asia and Latin America.^7^, ^8^, ^9^ For the age group 13–14 years, data showed prevalence values ranging from 0.2% in China to 24.6% in Columbia.^7^, ^8^, ^9^ A prevalence higher than 15% was found Africa, Latin America, Europe, and Oceania.^7^ AD prevalence appears to have reached a plateau in some countries such as the United Kingdom and New Zealand; on the other hand it seems to increase in low‐income countries such as Latin America or Southeast Asia, mostly in children compared with adolescents.^9^, ^10^ Environmental and epigenetic factors have been suggested as possible drivers of change in disease epidemiology.^7^, ^8^, ^9^

### 1.2 Pathogenesis

AD pathophysiology is multifactorial and involves complex interactions between genetic disorders, epidermal barrier defects, altered immune response, and microbiome changes.^3^, ^10^, ^11^ The role of genetics was firstly described as a higher occurrence of AD in children of parents with a history of atopic diseases.^12^, ^13^ Furthermore, loss of function mutations in filaggrin and other proteins of cornified envelope, such as loricrin, involucrin, and small proline‐rich proteins, is implicated in AD development due to alteration of the skin's barrier function, increased trans‐epidermal water loss, pH alterations, and dehydration.^14^, ^15^ One or more FLG mutation have been encountered in the 16–44% of individuals with moderate to severe AD, while FLG loss‐of‐function mutations are reported to be 7% to 10% in European patients.

The imbalance between T‐helper Th2/Th22 and Th1/Th17 can create alterations in cell‐mediated immune responses that occur concomitantly in the development of AD.^16^, ^17^

A distinction can be made; on the one hand, the onset of acute AD lesions may be associated with a significant increase in gene expression levels of interleukins Th2 (IL)‐4, IL‐13, IL‐31, and Th22 (i.e., IL‐22); on the other hand, in chronic skin lesions, a Th1/Th17 response may be observed along with an intensification of Th2 and Th22 responses.^16^, ^17^

There is an increasing data concerning different endotypes depending on ethnic or age. Th2 and Th22 lineage responses with lower Th1 and Th17 dominate in the European and American populations.^18^ In the Japanese population, there is increased frequency of the Th17 axis (and related IL‐17A, IL‐19, IL‐22, and S100A12) and suppression of the Th1 axis.^17^ In the Chinese population, in addition to Th2 activation, there is increased Th17/IL‐23 and increased expression of Th22‐induced markers.^17^, ^18^ AD African Americans showed targeted responses to Th2 and Th22 and a parallel attenuation of Th1/Th17 branch.^17^, ^18^

Furthermore, the skin endophenotype of pediatric AD is substantially different from that of adult/adolescent AD.^19^ Indeed, lesional skin children showed comparable or greater epidermal hyperplasia and cellular infiltration than adults with AD. Like adults, strong activation of the Th2 and Th22 axes and some Th1 skewing were present.^19^ However, pediatric AD is characterized by a significantly higher levels of Th17 related cytokines, Th9/IL‐9, IL‐33, and innate markers than adults.^19^ These features are responsible of phenotypic similarities to psoriasis.^18^

Finally, tymic stromal lymphopoietin (TSLP) is a central mediator of the skewed Th2 immunomodulation in the skin mediated via dendritic cells and the induction of mast cells and natural killer T cells. Also, the IL‐33 is upregulated in AD and seems involved in facilitating the typical Th2 cytokine profile that is a hallmark of AD inflammation. In AD patients, both IL‐31, and IL‐33 serum levels were higher in children than in adults.

## 2 METHODS

The authors followed criteria established in the preferred reporting items for systematic reviews and meta‐analyzes (PRISMA) guidelines for this review.^20^ A search of the Pubmed, Embase, and Cochrane Skin databases and that of clinicaltrials.gov was performed (until May 1, 2022). The search terms were “atopic dermatitis,” “children,” “diagnosis,” “differential diagnosis,” “diagnostic criteria,” “pathophysiology,” and “phenotypes”. Only English‐language publications were selected. Then, a revision of the abstracts and texts of the articles was made independently by each author. As a result, a total of 40 studies were selected.

## 3 DIAGNOSIS

### 3.1 Clinical features

Eczematous lesions typically show an age‐related distribution.^1^ Infants (≤2 years) show acute lesions, characterized by itchy papules and vesicles (raised lesions <1 cm), often associated with serous exudate and crusts.^1^ Typically, these lesions show a poorly defined erythema and involve the face, and trunk, the extensor surfaces of the limb, and sometimes the nappy area. In childhood (aged two years and older), AD is characterized by the appearance of dry skin, paler erythema, and lichenified papules and plaques affecting flexor surfaces, hands, and feet.^1^ Facial involvement is less prominent, but when present, a perioral, and periorbital distribution is observed.^1^ However, from age one to two years and onwards, polymorphous manifestations with different types of skin lesions are seen, such as nummular eczema or morphological variants including follicular type characterized by densely aggregated follicular papules.^1^

Adolescents and adults typically present symmetrical papules and plaques, often associated with lichenification and excoriations.^1^ The skin areas predominantly involved are flexural regions, face, neck, and distal extremities.^1^ Furthermore, in adults, involvement of the hand, nipple, or eyelid is more frequent. Generally, in adults the disease is more severe in persistent than late‐onset (>18 years) cases.^1^ Adult AD can present with variable clinical features, even atypical.^1^, ^6^ Indeed, Several AD phenotypes have been identified in adults, since the disease can present with atypical clinical features.^1^, ^6^, ^21^, ^22^

In adults and adolescents besides flexural eczema, head and neck eczema and hand eczema, which are the most frequent clinical phenotypes (84.9% and 84.2%, respectively), there are also other possible presentation such us portrait‐like dermatitis (20.1%), diffuse eczema (6.5%), eczema nummulare‐like (5.8%), prurigo nodularis‐like (2.1%), and erythrodermia (0.7%).^23^

### 3.2 Diagnostic criteria

AD can be difficult to define for the heterogeneity of clinical aspects, severity, and course.^24^ Currently, no definitive biomarkers exist for the diagnosis of AD. Several diagnostic criteria have been developed to support diagnosis. In 1980, Hanifin and Rajka proposed the first widely used diagnostic criteria for AD consisting of four major ([i] pruritus; [ii] typical morphology and distribution; [iii] chronic or chronically relapsing dermatitis; [iv] personal or family history of atopy) and 23 minor criteria.^24^ The diagnosis of AD requires three major features and three minor features.^25^ In 1997, the UK diagnostic criteria for AD were introduced by Williams et al.^26^ These criteria are adapted from those by Hanifin and Rajka and consist of one mandatory (pruritus) and five major criteria ([i] onset under the age of 2 years; [ii] a history of flexural involvement; [iii] a history of asthma or hay fever (or a history of atopic disease in siblings and parents if the child is under four years); [iv] a history of generally dry skin in the last year; and [v] visible flexural dermatitis).^26^ AD diagnosis requires mandatory and three or more of major criteria.^26^ Finally, the American Academy of Dermatology consensus criteria consist of useful clinical findings for clinicians, divided primarily into three categories^27^, ^28^:: (i) essential features (must be present) involving pruritus and acute or chronic eczematous dermatitis with typical morphology, age‐specific patterns, and chronic or relapsing history; (ii) important features (supports the diagnosis): early age of onset, personal or family history of atopy, and xerosis; (iii) associated features (nonspecific but suggest the diagnosis of AD): atypical vascular responses, keratosis pilaris/pityriasis alba/hyperlinear palms/ichthyosis, ocular/periorbital changes, other regional findings (eg, perioral changes/periauricular lesions), perifollicular accentuation/lichenification/prurigo.^27^, ^28^ However, these criteria are not always usable in a clinically variable disease such as AD. Indeed, they currently used mainly in clinical trials or for epidemiologic studies and not always in the everyday clinical practice Therefore, most guidelines and position papers consider the clinician's assessment as the gold standard for the diagnosis.^29^

## 4 DIFFERENTIAL DIAGNOSIS

**FIGURE 1:**
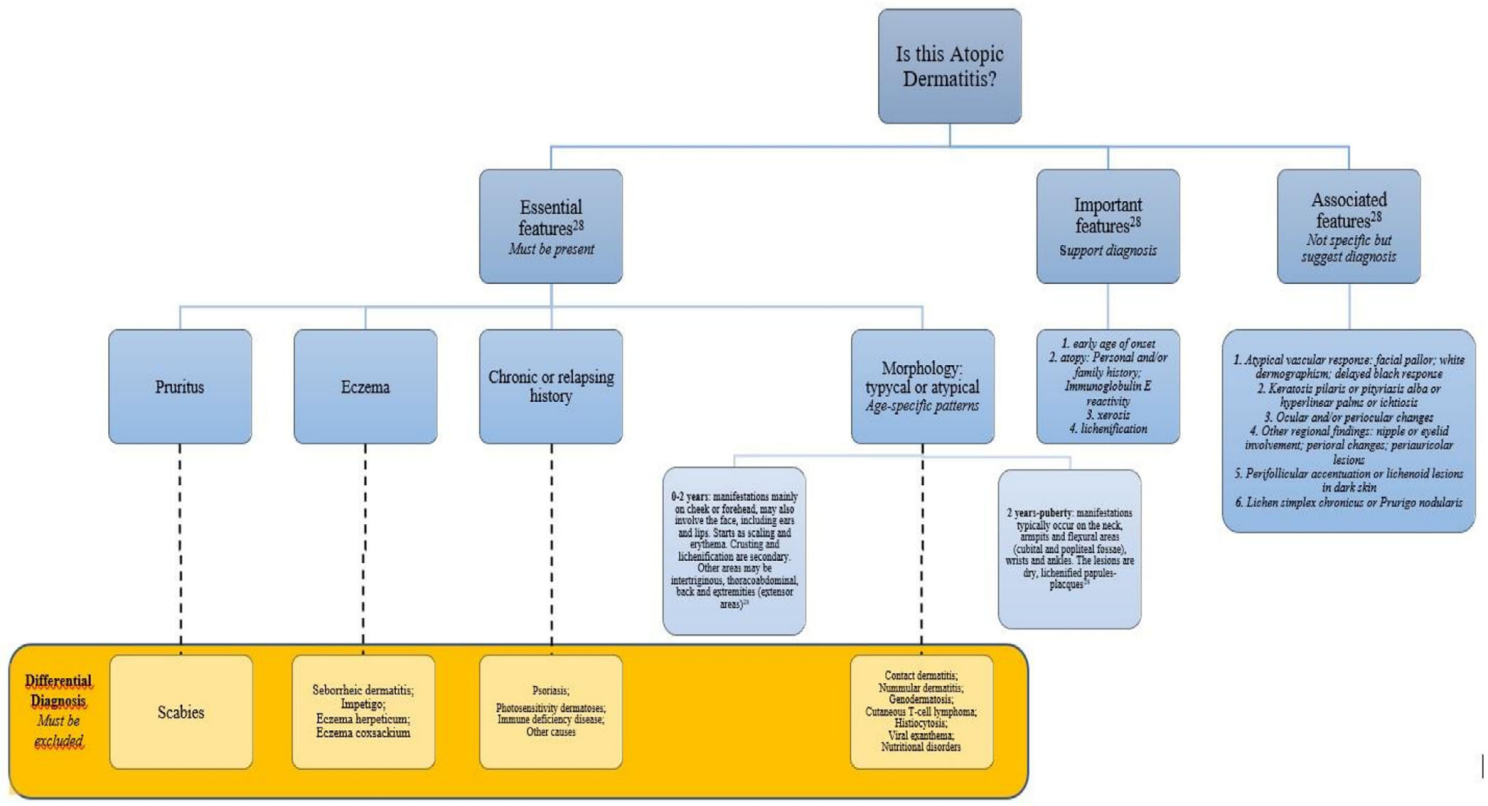
Algorithm for the diagnosis and differential diagnoses for children with AD

Although the diagnosis of typical AD of the infant affecting the face or the child with flexural involvement may not be difficult, several skin conditions can mimic AD. Then, laboratoristicand genetic workup is necessary in patients with chronic and unresponsive to treatment of eczematous dermatitis, especially if there are signs and symptoms suggestive of systemic disorders or syndromes.^30^ In Figure 1 we propose a diagnostic algorithm for the diagnosis and differential diagnoses for children with AD.

### 4.1 Inflammatory skin conditions

#### 4.1.1 Seborrheic dermatitis

Infantile seborrheic dermatitis (ISD) is a skin inflammatory condition, typically having its onset in the first weeks of life, with greasy crusts on the scalp, with a possible erythematous background, also called cradle cap.^31^, ^32^ Sometimes, it appears on the infant's face, especially around the eyes and nose area.^31^, ^32^ In early childhood it is often difficult to distinguish the two diseases due to the common sites of presentation (Table 1).^31^, ^32^ Indeed, both diseases can appear in the first eight weeks of life, typically at the scalp and forehead.^29^, ^32^, ^33^

**TABLE 1:** Differential diagnosis between infantile seborrheic dermatitis (ISD) and atopic dermatitis (AD)

Differential diagnosis		
	ISD	AD
Onset	< 2–10 weeks	< 1 years
Site	Intertriginous area (axillae and inguinal folds), eyebrows, retroaruicular folds, nasolabial folds, neck	Flexural involvement (antecubital and popliteal fossae), cheeks, periorificial
Appearance	Yellow greasy scale, Salmon‐pink poorly circumscribed patch^29^	Fine dry scale, red poorly circumscribed patch^29^
Symptoms	Absent itch	Severe itch

Classically, they are considered as separate entities, but many authors believe they are part of a single pathological continuum.^34^, ^35^ In a study by Alexopoulos et al.^35^ 87 children were examined and diagnosed with typical clinical features of ISD between 1997 and 2011. Forty‐nine of the 87 children were followed up over a period of 5 years. Among these, 30/87 (34.4%) children with ISD developed AD: 23 of them were diagnosed with only AD, at an average time interval of 6.4 months from ISD onset, while seven presented with clinical features of AD at the time of ISD diagnosis.

#### 4.1.2 Psoriasis

Pediatric psoriasis (PP) can be misdiagnosed as AD, since it presents with finer and less defined plaques and less white desquamation than the adult and because AD is more frequent in childhood.^29^, ^36^ Moreover, it should be noted that about 5% of the pediatric population has an overlap of AD and psoriasis.^37^ However, established criteria exist for AD, but not for PP. Although histology is diagnostic, it is rarely practical in children.^1^ Diagnosis has therefore clinical, but difficulties can arise from the subtle presentation of PP.^38^ The most common form of psoriasis in childhood is inverse psoriasis, commonly confused with nappy dermatitis.^31^ Psoriasis might be hypothesized if a dermatitis is particularly resistant to treatment.^31^ In Table 2 the main clinical characteristics of both AD and psoriasis are reported.

**TABLE 2:** Differential diagnosis between psoriasis and atopic dermatitis (AD)

Differential diagnosis		
	Psoriasis	AD
Onset	All ages	All ages
Site	Extensor area, diaper area, gluteal cleft, uncommon on face	In infants, AD is located on the face and the extensor surfaces of the limbs. From age 1–2 years and onwards, flexural involvement (antecubital and popliteal fossae), periorificial involvement
Appearance	Rare white scale, pink thick and sharply circumscribed patch^37^	Fine dry scale, red poorly circumscribed patch^16^
Symptoms	Mild–moderate itch	Severe itch

#### 4.1.3 Nummular dermatitis or nummular eczema

Nummular dermatitis (ND) is a chronic dermatitis with morphologically coin‐shaped lesions, is idiopathic and is not associated with a history of atopy.^29^, ^32^ On the other hand, ND‐like lesions are sometimes found in AD. However, ND rarely occurs before the age of five years, is not associated with other AD features, and does not persist into adolescence and adulthood.^28^, ^29^, ^39^

#### 4.1.4 Contact dermatitis

Allergic contact dermatitis (ACD) is a type IV‐delayed hypersensitivity reaction and should be considered in differential diagnosis with AD as well as co‐existing with it and/or exacerbating it.^30^ It presents as a demarcated, erythematous plaques with vesiculations in the acute phase, or xerotic crusts in the chronic phase.^31^ In AD patients with persistently and refractory to therapies dermatitis, ACD should be taken into consideration and patch test performed.^30^, ^32^ A recent study suggested a consistent prevalence of contact sensitizations among children, with higher sensitivity rate among children with AD than without AD.^40^ In this study, the most frequent contact allergens reported were nickel sulfate, cobalt chloride, methylisothiazolinone, fragrance mix‐2, potassium dichromate, fragrance mix‐1, and methylchloroisothiazolinone/methylisothiazolinone.^40^

Irritant CD is caused by skin injury, direct cytotoxic effects, or cutaneous inflammation from contact with an irritant.^39^ Symptoms may occur immediately and may persist if the irritant is unrecognized.^39^ Recognition of isolated CD relies on temporal pattern, as well as suggestive distribution.^39^ In pediatric patients, irritant CD is most common on the face, dorsal aspect of the hands, and “diaper area,” often triggered by frequent cycles of skin wetting and drying as well as exposure to endogenous (e.g., drool, lip‐licking, urine, and feces) or exogenous (e.g., cleansing products, highly alkaline or acidic foods) irritants.^39^

### 4.2 Infections and infestations

Several factors can contribute to the increased infections in AD such as skin barrier defects, suppression of cutaneous innate immunity by type 2 inflammation, Staphylococcus aureus colonization, and cutaneous dysbiosis.^41^ Furthermore, some infections or infestations can mimic AD.^41^

#### 4.2.1 Impetigo

Impetigo is a superficial bacterial infection caused mainly by Staphylococci and Streptococci, and takes the form of pain, erythema, and serum scabs.^39^ It may therefore be confused with AD or coexist with it.^39^ Typically, it presents with oozing serum that has dried up, giving it a honey‐crusted appearance surrounded by an erythematous base.^42^ Impetiginous lesions may also present with fluid‐filled blisters (bullous impetigo).^39^ Furthermore, impetigo can occur in eczema‐ affected skin that's open and “weepy.”^39^ People with AD are more likely than the general population to have colonized *Staphylococcus aureus*, leaving them more prone to infections.^39^

#### 4.2.2 Molluscum dermatitis

Molluscum contagiosum (MC) is a viral infection of the skin, especially in children.^29^, ^31^, ^39^, ^41^ It is found more frequently in individuals with AD, but there is not necessarily an association.^29^, ^31^, ^39^, ^41^ Skin barrier defects predispose patients with AD to MC, and long‐term scratching leads to the spread by autoinoculation. MC infection in AD has been associated with FLG mutations.^29^, ^31^, ^39^, ^41^

The diagnostic doubt arises when an itchy eczema (molluscum dermatitis) develops around the molluscum. However, a careful objective examination will highlight the papule of MC.^29^, ^31^, ^39^, ^41^

#### 4.2.3 Eczema herpeticum

Eczema herpeticum (EH) is a life‐threatening viral infection, which occurs acutely and is due to HSV in patients with a history of AD.^39^, ^41^ Nearly a third of children who are hospitalized for AD infectious complications were related to EH.^39^, ^41^ It can manifest with skin pruritus or pain and presence of vesicles, punched‐out erosions, or haemorrhagic crusts that can become more extensive.^39^, ^41^ A local skin infection may progress to disseminated vesicles with skin breakdown. Systemic EH infection may present with fever, malaise, viremia, and complications including kerato‐conjunctivitis, encephalitis, and septic shock.^39^, ^41^ Patients with AD who develop EH tend to have more severe AD, earlier‐onset AD, high total serum immunoglobulin E/peripheral eosinophils, and presence of other atopic diseases such as food allergies and asthma, as compared to their AD counterparts without EH.^39^, ^41^

It may be confused with impetigo, especially in patients with a history of severe AD (EH incognito) or in the early stages with AD itself.^39^, ^41^ Bacterial positivity on skin culture does not exclude EH. The diagnosis can be confirmed by PCR or Tzank smear.^39^, ^41^

#### 4.2.4 Eczema coxsackium

Coxsackium eczema (EC) is due to coxsackievirus A6 and frequently presents as EH‐like with a greater prevalence of haemorrhagic vesicles over dermatitis, thus casting diagnostic doubt with AD, which is often associated.^39^, ^41^ Some patients with EC may also have symptoms of the hand‐foot‐mouth disease, such as oral sores and papules involving hands and feet.^39^, ^41^ Other possible symptoms include fever, sore throat, and poor appetite.^39^, ^41^ EC should be considered also as a differential diagnosis for EH because it can present with extensive vesicles and skin erosion.^39^, ^41^ However, contrast to EH, EC is not life‐threatening and can be managed with standard AD treatments.^39^, ^41^ The diagnosis is supported by PCR.^39^, ^41^

#### 4.2.5 Viral exanthem

Viral exanthems (e.g., hand‐foot and mouth disease, unilateral laterothoracic exanthema, Gianotti‐Crosti syndrome) are a heterogeneous group of disorders with cutaneous manifestations that may occasionally resemble AD.^39^, ^43^ However, the sudden, symmetrical mode of onset with small, well‐demarcated plaques allow them to be distinguished from AD, in addition to the presence of other systemic symptoms.^39^, ^43^

#### 4.2.6 Candidiasis

In nappy eczema, the diagnosis may include AD, ACD but also Candida, which often also acts as a cofactor.^39^, ^44^ Of note is congenital candida, which has burn‐like manifestations, with desquamation, which can make diagnosis difficult.^39^, ^44^

#### 4.2.7 Tinea corporis and capitis

Although the classic appearance of tinea corporis is an erythematous ring‐shaped plaque with central resolution, atypical morphology is not uncommon and may lead to suspicion of AD, ND or pityriasis rosea.^29^, ^31^, ^32^, ^39^, ^43^ Tinea capitis, on the other hand, when presenting with fine non‐inflamed plaques, may resemble ISD or AD.^28^, ^30^, ^31^, ^41^, ^42^ However, the presence of alopecia, pustules, lymphadenopathy, and broken hair may help the diagnosis.^29^, ^31^, ^32^, ^39^, ^43^ Microscopic examination is a useful tool, but the presence of hyphae does not necessarily exclude AD.^29^, ^31^, ^32^, ^39^, ^43^

#### 4.2.8 Scabies

Because of the severe itching and eczematous dermatitis involved, scabies is among the differential diagnoses of AD.^29^, ^31^, ^32^, ^33^, ^39^, ^43^ However, scabies itching is localized mainly to the armpits and groin in children, as well as to the interdigital spaces, which are not typical sites for eczema.^29^, ^31^, ^32^, ^33^, ^39^, ^43^ A positive history of itching, especially nocturnal itching, in other family members helps the diagnosis.^29^, ^31^, ^32^, ^33^, ^39^, ^43^ The dermoscopic examination looking for the mite and the burrow confirms the diagnosis.^29^, ^31^, ^32^, ^33^, ^39^, ^43^

### 4.3 Neoplasm

Hypopigmented mycosis fungoides (MF) is the most common childhood variant of cutaneous T‐cell lymphoma (CTCL); it may resemble pityriasis alba found in AD patients.^30^, ^32^, ^44^ Indeed, AD accounts for almost one‐third of the initial diagnoses of MF.^30^, ^32^ It presents as ill‐defined hypopigmented and minimally desquamating patches distributed mainly in the photo‐protected areas (bathing suits area), which may be itchy and finely wrinkled due to atrophy.^30^, ^41^, ^44^ The gold standard of diagnosis is biopsy, which will show a lymphocytic infiltrate with marked epidermotropism, and an immunohistochemical examination and T‐cell rearrangement study may be useful.^32^

Langerhans cell histiocytosis (LCH) is also in differential diagnosis.^30^, ^32^, ^44^ LCH on the skin has a heterogeneous presentation, but the most typical is with flesh‐colored papules and plaques on the folds, scalp, and lower trunk.^31^ Petechiae are found on seborrheic areas, making the differential diagnosis with ISD and AD easier.^31^ Another differential is the unresponsive nature of the classic AD therapies.^43^ Letterer‐Siwe disease (LSD) is a malignant form of LCH most seen in children with a clinic resembling ISD or more frequently with ulceration of the scalp and folds.^39^ Petechiae and pustules are also reported.^39^

### 4.4 Genetic diseases

#### 4.4.1 Hyperimmunoglobulin E syndromes

A very high IgE title is frequently found in AD patients, which often poses the problem of differential diagnosis with hyper‐IgE syndrome (HIES).^33^, ^45^ This is due to hyperactivation of the Th2 arm typical of AD, which leads to an increase in IgE.^45^ The term HIES often refers to a group of syndromes that have genetic defects that lead to an increase in IgE, such as mutations in inflammatory cytokine receptors, components involved in the rearrangement of the cytoskeleton or glycosylation (Table 3).^45^

**TABLE 3:** Main genetic syndromes ranging in differential diagnosis with atopic dermatitis

Syndrome	Defect	Distinctive features associated with AD	References
Autosomal dominant hyper‐IgE syndrome	STAT3: abnormal cytokine signaling	Hyper‐IgE, pneumatoceles and bacterial pneumonia, absence of Th17	43
Autosomal recessive hyper‐IgE syndromes	DOCK8: cytoskeletal dysfunction	Hyper‐IgE, viral infection susceptibility (eczdema herpeticum or molluscum)	28, 43, 44
Autosomal recessive hyper‐IgE syndromes	PGM3: glycosylation disorder	Hyper‐IgE, neurological and bone atypia, leukopenia, increased Th17	43
Wiskott‐Aldrich syndrome (X‐linked recessive)	WAS: cytoskeletal dysfunction	Possible hyper‐IgE, haemorrhagic diathesis, thrombocytopenia, progressive combined immunodeficiency (recurrent infections)	30, 31, 43
IPEX syndrome	FOXP3: absent Tregs	Hyper‐IgE, eosinophilia, diarrhea, endocrine autoimmunity	43
Omenn syndrome	RAG1/2: oligoclonal T cells; lymphopenia	Hyper‐IgE, eosinophilia, SCID with few T cells present, erythrodermia,	43
Netherton syndrome	SPINK5‐LEKTI: inappropriate protease activation	Hyper‐IgE, eosinophilia, ichthyosis, erythrodermia, marked atopy, trichoresis invaginate (bamboo hair), alopecia, chronic diarrhea, growth retardation	29, 39, 44, 45
Peeling skin syndrome type B	CDSN: compromised cell adhesion	Hyper‐IgE, ichthyosis, erythrodermia, severe pruritus, thin hair	44
SAM syndrome	DSG1: compromised cell adhesion	Hyper‐IgE, multiple allergies and metabolic wasting, erythrodermia, hypotrichosis, growth retardation	44

Abbreviations: CDSN, corneodesmosin; IPEX, immunodysregulation polyendocrinopathy enteropathy X‐linked; SCID, severe combined immunodeficiency.

*Source*: Adapted from Lyons et al.^45^

Phenotypically, they present with eczematous rashes resembling AD.^45^ However, they present from the first weeks of life, which is atypical for AD.^45^

An immunodeficiency syndrome must be hypothesized when chronic eczematous dermatitis is associated with secondary infections or other specific signs.^30^ Thus, the correct workup in suspecting such diseases includes growth curve assessment, targeted laboratory and imaging tests, and genetic testing.^39^, ^45^ It is important to emphasize that the mere finding of elevated IgE levels does not entitle the patient to be immediately classified as HIES.^30^, ^45^, ^46^ In fact, HIES patients often have even lower levels of IgE than AD patients.^30^ Therefore, the laboratory data necessarily require an overall clinical correlation: allergies, severe and recurrent infections, eosinophilia, and obviously skin signs must be investigated.^45^, ^46^

## 5 THERAPEUTIC APPROACH

AD strongly impacts the quality of life of young patients as well as their families .^46^, ^47^ A recent analysis found a prevalence of severe AD in about 8% of children between 6 and 11 years of age.^48^ The treatment of AD is very difficult in this population due to both the age and severity of the disease.^49^ In fact, in the pediatric population, treatment mostly consists of topical emollients and corticosteroids (TCs).^49^ However, TCs are not easy to use in children as they have a higher absorption of the compound due to their reduced weight in relation to body surface area, inducing both cutaneous and systemic side effects (e.g., suppression of the hypothalamic–pituitary–adrenal axis).^50^ Alternatives include topical calcineurin inhibitors (TCIs), tacrolimus and pimecrolimus, especially for sensitive areas, such as the face and folds.^50^

The Italian guidelines for AD recommend proactive therapy (twice weekly) with tacrolimus because they have observed that it reduces the time to relapse.^51^

However, there are restrictions due to both age (they are contraindicated <2 years) and high cost.^49^ In case of uncontrolled disease, systemic corticosteroids (SCs) can be used, however they are not preferred due to the unfavorable risk–benefit ratio in pediatric age.^52^ Other immunosuppressants such as cyclosporine, methotrexate or azathioprine, although effective, are off‐label in children and potentially systemically toxic.^52^

The European Medicines Agency (EMA) recently approved dupilumab in the 6–11 age group for children with moderate–severe AD not controlled by topical therapies.^52^ It is a fully human monoclonal antibody that inhibits the alpha subunit of the IL‐4 receptor, which is shared by both IL‐4 and IL‐13, key cytokines in the pathogenic pathway of AD.^52^, ^53^ Recent studies showed that the cytokine profile in the pediatric and adolescent population differs from that of adults, with a polarization toward the Th2 and 17 axes and the absence of Th1 upregulation.^54^, ^55^ Therefore, there is a clear need to act as early as possible in childhood with immunomodulatory drugs, such as dupilumab, to prevent AD chronicity and developing associated diseases.^54^

## 6 CONCLUSION

AD is one of the most common infant skin diseases, affecting more than 20% of children in industrialized areas: 45% of AD cases occur before six months of age, 60% before the first year of life, 89% before the age of five years.^47^, ^49^, ^50^, ^56^ The diagnosis can be easy due to typically distributed eczematous lesions, albeit with age‐related differences.^1^ However, it is also extremely heterogeneous in severity, course, and sometimes particular clinical features.^1^ Currently, no diagnostic criteria more efficacious than an expert dermatologist exist for the diagnosis of AD. Misdiagnosis and delayed treatment will have an impact not only on the child's physical health but also and above all on psychological health. Episodes of anxiety, depression, isolation, and even bullying are not uncommon, with stigmatization of the pathology and negative consequences on the child's future education and productivity.^56^, ^57^, ^58^, ^59^, ^60^

Therefore, it is important to identify AD from the early stages of life and choose the most suitable therapeutic approach for the patient, also in anticipation of the evolution of the disease in adult age.

## AUTHOR CONTRIBUTIONS

Maddalena Napolitano conceptualization, validation, visualization, writing‐original draft preparation, writing ‐ review & editing. Gabriella Fabbrocini conceptualization, validation, visualization, writing‐original draft preparation, writing ‐ review & editing. Fabrizio Martora conceptualization, validation, visualization, writing‐review & editing, supervision. Lucia Genco validation, visualization. Matteo Noto conceptualization, validation, visualization, writing‐review & editing, supervision. Cataldo Patruno conceptualization, validation, visualization, writing‐original draft preparation, writing ‐ review & editing.

All authors read and approved the final version of the manuscript.

## CONFLICT OF INTEREST

All the authors declare that have no conflict of interest.

## ETHICS STATEMENT

Not required.

## Data Availability

The data that support the findings of this study are available from the corresponding author upon reasonable request.
